# Epidemiologic Changes of Scrub Typhus in China, 1952–2016

**DOI:** 10.3201/eid2606.191168

**Published:** 2020-06

**Authors:** Zhongjie Li, Hualei Xin, Junling Sun, Shengjie Lai, Lingjia Zeng, Canjun Zheng, Sarah E. Ray, Nicole Davis Weaver, Liping Wang, Jianxing Yu, Zijian Feng, Simon I. Hay, George F. Gao

**Affiliations:** Chinese Center for Disease Control and Prevention, Beijing, China (Z. Li, H. Xin, S. Lai, L. Zeng, C. Zheng, L. Wang, J. Yu, Z. Feng, G.F. Gao);; Qingdao City Center for Disease Control and Prevention, Shandong, China (H. Xin);; University of Southampton, Southampton, UK (S. Lai);; University of Washington, Seattle, Washington, USA (S.E. Ray, N.D. Weaver, S.I. Hay);; Chinese Academy of Sciences, Beijing (G.F. Gao)

**Keywords:** scrub typhus, incidence, trends, seasons, China, surveillance, *Orientia tsutsugamushi*, bacteria, vector-borne infections, zoonoses

## Abstract

Scrub typhus, a miteborne rickettsiosis, has emerged in many areas globally. We analyzed the incidence and spatial–temporal distribution of scrub typhus in China during 1952–1989 and 2006–2016 using national disease surveillance data. A total of 133,623 cases and 174 deaths were recorded. The average annual incidence was 0.13 cases/100,000 population during 1952–1989; incidence increased sharply from 0.09/100,000 population in 2006 to 1.60/100,000 population in 2016. The disease, historically endemic to southern China, has expanded to all the provinces across both rural and urban areas. We identified 3 distinct seasonal patterns nationwide; infections peaked in summer in the southwest, summer-autumn in the southeast, and autumn in the middle-east. Persons >40 years of age and in nonfarming occupations had a higher risk for death. The changing epidemiology of scrub typhus in China warrants an enhanced disease control and prevention program.

Scrub typhus is a life-threatening disease caused by *Orientia tsutsugamushi*, an obligate intracellular bacterium transmitted by the larvae of trombiculid mites (*1*). Only biting larvae of Asian scrub typhus chiggers (*Leptotrombidium* spp.) can transmit the disease. After the bite of an infective mite, a characteristic necrotic inoculation lesion (an eschar) can develop. The microorganism then spreads through the lymphatic fluid and blood, causing manifestations including fever, headache, rash, lymphadenopathy, and mental changes (*1*). Without appropriate treatment with specific antimicrobial drugs (e.g., tetracycline, chloramphenicol, doxycycline, or azithromycin), >6% of infected patients will die (*2*,*3*). There is no licensed human vaccine to prevent scrub typhus infection.

Globally, scrub typhus is traditionally regarded as a disease endemic to a region called the Asia-Pacific tsutsugamushi triangle, which extends from Pakistan in the west to far eastern Russia in the east to northern Australia in the south. In some countries of Southeast Asia, scrub typhus is a leading cause of treatable nonmalarial febrile illness (*4**–**6*). It is estimated that scrub typhus threatens >1 billion persons, causes at least 1 million clinical cases per year, and is associated with substantial mortality rates globally (*1*,*7*). The 2010s saw a widespread reemergence of scrub typhus in endemic regions such as India, Korea, Laos, and the Maldives (*3*,*6*,*8*). The recent emergence of scrub typhus in the Arabian Peninsula, Chile, and possibly Kenya suggests wider global distribution of this disease in tropical and subtropical regions, far from the tsutsugamushi triangle (*9**–**12*). Although scrub typhus poses the greatest threat to residents of eastern and southern Asia as well as tourists traveling to these regions, it remains a neglected disease globally. The lack of both research and a nationwide surveillance system within many endemic regions have resulted in poorly understood epidemiologic characteristics and disease burden of scrub typhus at global, national, and subnational levels (*7*). The Global Burden of Disease Study publishes estimates for 333 diseases and injuries, but currently bundles scrub typhus with other neglected tropical diseases, rather than providing specific burden estimates (*13*).

In China, scrub typhus cases were recorded in the early 1950s, and a disease surveillance system for scrub typhus was established in 1952 (*14*,*15*). *Leptotrombidium deliense* and *L. scutellare* mites are the 2 principal vectors transmitting the disease in the country (*16*). *L. deliense* mites inhabit southern China and emerge in April, peaking in June–August, and decreasing September–December, whereas *L. scutellare* is widespread in China, emerging annually in October–December, and is the main mite species in northern China (*17*). Several studies have revealed that the incidence of scrub typhus is rising nationwide (*18*,*19*); however, the changing epidemiologic characteristics of scrub typhus at the subnational level and among the subgroups are not fully illustrated. After the SARS outbreak in 2003, a nationwide population-based infectious disease surveillance system was developed in China to collect epidemiologic information at the patient level, enabling study of the epidemiologic features of a disease at the smaller scale of a geographic region and specific population (*20*,*21*).

In this study, we systematically collated longitudinal nationwide surveillance data of scrub typhus in China from 1952–1990 and 2006–2016. We explored changes in the disease’s epidemiologic characteristics, both spatiotemporally and seasonally, and changes in high-risk population groups at both national and subnational levels.

## Materials and Methods

### National Surveillance of Scrub Typhus

In China, the national surveillance program on scrub typhus (Appendix Tables 1, 2) varied through 3 time periods. Initially, mail-based reporting of monthly aggregated data occurred in 1952–1989. Beginning in 1952, data on scrub typhus were reported on a voluntary basis in certain areas of China. In 1955, scrub typhus became a statutorily notifiable disease, and reporting all suspected, probable, and laboratory-confirmed scrub typhus cases was required. Reporting was suspended during 1990–2005; in 1990, scrub typhus was removed from the list of notifiable diseases, due to its relatively low threat. Therefore, nationwide data on the disease are not available from 1990–2005. Internet-based reporting of individual cases occurred in 2006–2016; in 2006, scrub typhus was added as a reporting disease in the National Notifiable Infectious Disease Reporting Information System at the Chinese Centers for Disease Control and Prevention (China CDC). The information on individual cases includes patient demographic information (name, sex, occupation, residence), date of illness onset, case classification (confirmed, probable, suspected), date of diagnosis, date of report, reporter institution, and date of death (if applicable).

The National Health Commission of the People’s Republic of China determined that the collection of data on human cases of scrub typhus was part of a routine public health investigation and was exempt from institutional review board assessment. All data in this study are anonymous so that individual patients cannot be identified.

### Case Definition

Scrub typhus cases have been classified as probable (clinically diagnosed) or confirmed (laboratory confirmed) in accordance with the diagnosis criteria and case classification guidelines issued by the Chinese national health authorities (Appendix Table 2). Probable cases are diagnosed by local experienced physicians according to epidemiologic exposure (travel to a disease-endemic area and contact with chiggers or rodents <3 weeks before the onset of illness) and clinical manifestations (such as high fever, lymphadenopathy, skin rash, and eschars or ulcers). Confirmed cases are probable cases with 1 positive result among the following tests: Weil-Felix test, indirect immunofluorescence antibody assay, PCR, or isolation of *Orientia tsutsugamushi* (*22*).

### Data Analysis

Our analysis comprised all probable and confirmed human scrub typhus cases in 1952–1989 and 2006–2016 (Appendix Tables 3, 4). We calculated the annual incidence rate by dividing the number of human scrub typhus cases by the corresponding population at the end of a given year. We obtained population data used to calculate incidence rates from the National Bureau of Statistics of China (*23*). We calculated the case-fatality ratio by dividing the number of human scrub typhus deaths by the number of scrub typhus cases. We used Joinpoint regression models to examine the incidence trends from 1952–2016 and calculated annual percentage changes (*24*).

We created a heat map of yearly incidence rates (standardized range 0–1) to visualize the long-term change over the 49-year period by province (Appendix Table 5, Figure 3). To assess the changing spatial and temporal patterns of the affected areas from 2006–2016, we plotted the newly affected and previously affected counties and townships by year in both the rural and urban areas where each case resided. In our study, the rural areas refer to the village or rural town, and urban areas refer to the city community or urban town. We identified epidemiologically related regions of scrub typhus based on hierarchical cluster analysis with Ward’s minimum variance method, using the Pearson correlation coefficient matrix of average weekly cases between paired provinces as distance.

To quantify the characteristics of scrub typhus season, we fitted multiple linear regression models to time series of the weekly number of scrub typhus cases during 2006–2016, including harmonic terms representing annual and semiannual periodic cycles as described previously (*25**–**27*). We defined scrub typhus season as the period of consecutive weeks in which >95% of the total annual cases occurred and as the smallest number of consecutive weeks. We characterized scrub typhus season by plotting the season onset, peak (the week with the highest case number in the fitted seasonal curve), and offset against the calendar week of the year. We used a multivariable logistic regression model to explore the predictors of death for scrub typhus. We used R statistical software version 3.4.1 (https://www.r-project.org) to produce graphs and perform statistical analyses (*28*) and ArcGIS version 10.2.2 (ESRI, https://www.arcgis.com) to plot geographic patterns. We used Joinpoint version 4.5.0.0 (US National Cancer Institute, https://surveillance.cancer.gov/joinpoint) to examine incidence trends.

## Results

### Overall Incidence

**Figure 1:**
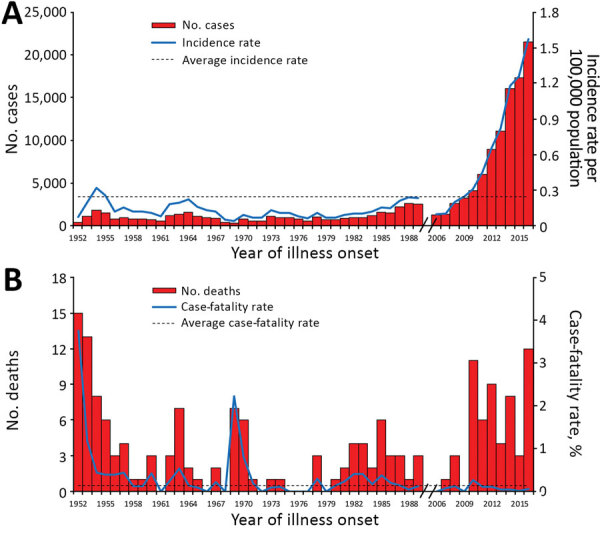
Reported cases and deaths of scrub typhus in China, 1952–2016. A) Aggregated number of cases by year (red bars), annual incidence rate (blue line), and average annual incidence rate (black dashed line) per 100,000 residents. B) Aggregated number of deaths by year (red bars), case-fatality ratio (blue line), and average annual case-fatality ratio (black dashed line). The data from 1990–2005 are missing because surveillance for scrub typhus was suspended during the period.

During the 49 years covered in this study (1952–1989 and 2006–2016), a total of 133,623 scrub typhus cases were recorded throughout the country. During 1952–1989, the occurrence of the disease remained at a stable, low level, with a mean annual incidence rate of 0.13 (95% CI 0.11–0.15) per 100,000 population (Figure 1, panel A). Since 2006, the annual incidence rate has increased sharply, changing from 0.09/100,000 population in 2006 to 1.60/100,000 population in 2016 (>16-fold increase) (Table 1), with an annual percent change of 32% (95% CI 30%–34%) (Appendix Figure 1).

**Table 1:** Characteristics of reported scrub typhus cases, China, 2006–2016*

Characteristic	2006	2007	2008	2009	2010	2011	2012	2013	2014	2015	2016
No. cases	1,254	1,340	2,613	3,237	4,088	6,020	8,928	11,111	16,035	17,293	21,562
No. probable cases	1,049	1,187	2,360	2,939	3,811	5,630	8,555	10,536	15,339	16,696	20,997
No. confirmed cases	205	153	253	298	277	390	373	575	696	597	565
Incidence†	0.09	0.10	0.19	0.24	0.30	0.45	0.66	0.82	1.19	1.28	1.60
Sex											
M	642 (51)	711 (53)	1,385 (53)	1,660 (51)	2,040 (50)	2,884 (48)	4,228 (47)	4,910 (44)	7,222 (45)	8,021 (46)	9,871 (46)
F	612 (49)	629 (47)	1,228 (47)	1,577 (49)	2,048 (50)	3,136 (52)	4,700 (53)	6,201 (56)	8,813 (55)	9,272 (54)	11,691 (54)
Age, y, median (IQR)‡	46 (27–58)	46 (27–59)	48 (30–60)	44 (25–57)	46 (28–59)	50 (33–61)	50 (36–62)	51 (38–62)	52 (39–63)	53 (42–64)	53 (41–64)
<5	86 (7)	92 (7)	198 (8)	279 (9)	358 (9)	423 (7)	606 (7)	638 (6)	775 (5)	681 (4)	928 (4)
5–14	143 (11)	139 (10)	173 (7)	341 (11)	344 (8)	401 (7)	539 (6)	629 (6)	811 (5)	716 (4)	899 (4)
15–44	373 (30)	402 (30)	783 (30)	1,012 (31)	1,200 (29)	1,543 (26)	2,147 (24)	2,589 (23)	3,683 (23)	3,738 (22)	4,578 (21)
45–60	397 (32)	415 (31)	878 (34)	1,001 (31)	1,320 (32)	2,035 (34)	3,169 (35)	4,038 (36)	5,791 (36)	6,339 (37)	7,797 (36)
>61	255 (20)	292 (22)	581 (22)	604 (19)	866 (21)	1,618 (27)	2,467 (28)	3,217 (29)	4,975 (31)	5,819 (34)	7,360 (34)
Residence§											
Rural	892 (76)	1,023 (79)	2,108 (83)	2,758 (87)	3,512 (88)	5,231 (88)	7,725 (87)	9,622 (87)	13,803 (87)	15,058 (88)	18,597 (89)
Urban	279 (24)	264 (21)	424 (17)	408 (13)	477 (12)	718 (12)	1,170 (13)	1,443 (13)	2,105 (13)	2,051 (12)	2,414 (11)
Occupation, farmer	733 (58)	859 (64)	1,700 (65)	2,079 (64)	2,721 (67)	4,250 (71)	6,179 (69)	7,853 (71)	11,842 (74)	13,230 (77)	16,670 (77)
No. in endemic province	16	19	18	22	20	20	23	21	27	26	25
No. in endemic county	222	225	327	372	431	509	582	692	769	809	883
No. in endemic township	616	683	1,183	1,283	1,523	2,088	2,644	3,264	4,125	4,353	5,025
No. deaths	0	1	3	0	11	6	9	4	8	3	12

*Values are no. (%) except as indicated. IQR, interquartile range.  †No. cases/100,000 population.  ‡Some columns do not add up to 100% because of rounding. §Some columns do not add up to equal the total because of missing data.

### Demographic Features

From 2006–2016, the mean age of case-patients was 48 years (range 1 month–98 years). The median age increased from 46 years (interquartile range 27–58 years) in 2006 to 53 years (interquartile range 41–64 years) in 2016. Overall, case-patients >45 years of age were the most common (66%); the highest incidence was among those 60–64 years (1.67 cases/100,000 population) and 65–69 years (1.71 cases/100,000 population). However, a subgroup of children <5 years of age had the highest incidence rate in the southwest disease-endemic regions (4.30 cases/100,000 population for Yunnan and Sichuan provinces combined) (Table 1). The sex ratio of male to female changed from 1.09:1 in 2006–2009 to 0.85:1 in 2010–2016. In general, farmers (73%) were the most affected group, with 86% of cases occurring in rural areas.

### Spatial-Temporal Distribution

**Figure 2:**
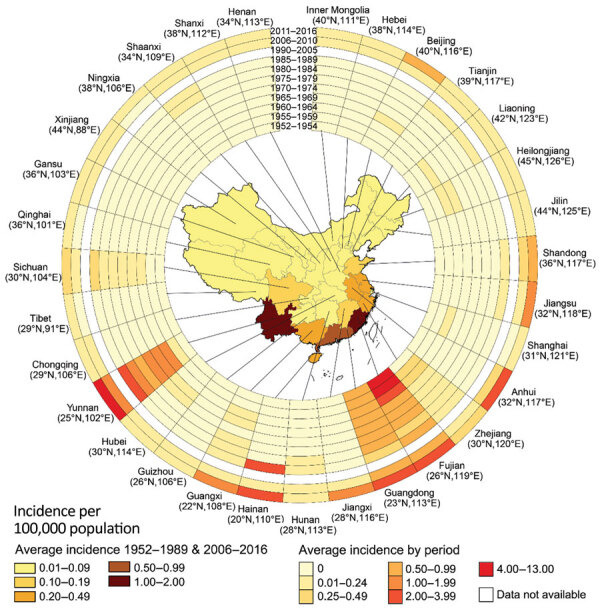
Incidence rate of scrub typhus for each province of China during 1952–1989 and 2006–2016, by time period. Annual average incidence of scrub typhus per 100,000 population in the 31 provinces investigated is shown. The rings contain data for 11 periods studied; the innermost ring shows data for early periods of 1952–1954, and the outermost ring data for 2011–2016. The latitude and longitude of the capital city of each province are shown.

**Figure 3:**
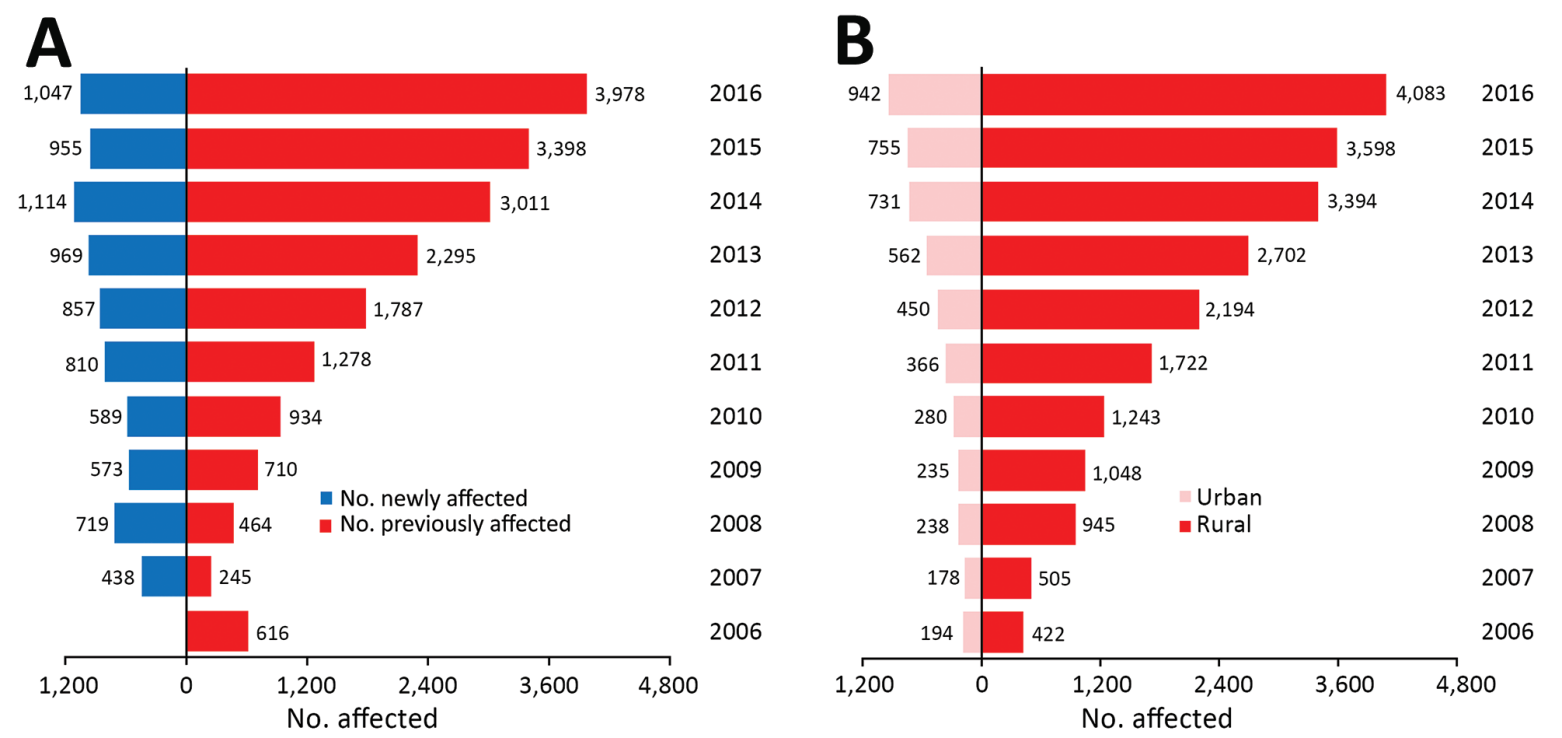
Number of townships with scrub typhus cases reported in China during 2006–2016, by year. A) Number of affected townships for each year, divided by those that were affected in previous years (red bars) and those newly affected townships for each year (blue bars). B) Total number of affected townships for each year in rural (red bars) and urban (light pink bars).

During the 28 years from 1952–1979, nearly all cases (99.9%) occurred in the southern provinces of China; sporadic cases were recorded in several northern provinces. Beginning in 1980, the disease began to expand slowly northward and westward; the number of affected provinces gradually increased from 17 during 1952–1989 to all 31 provinces during 2011–2016 (Figure 2). We saw dramatic expansion in both rural and urban areas. In rural areas, the affected townships increased nearly 10-fold, from 422 in 2006 to 4,083 in 2016, and the affected urban townships expanded from 194 in 2006 to 942 in 2016 (Figure 3). As of 2016, a total of 28% (883/3,101) of all counties and 11% (5,025/44,539) of all townships recorded cases of scrub typhus throughout the country (Appendix Figure 2).

**Figure 4:**
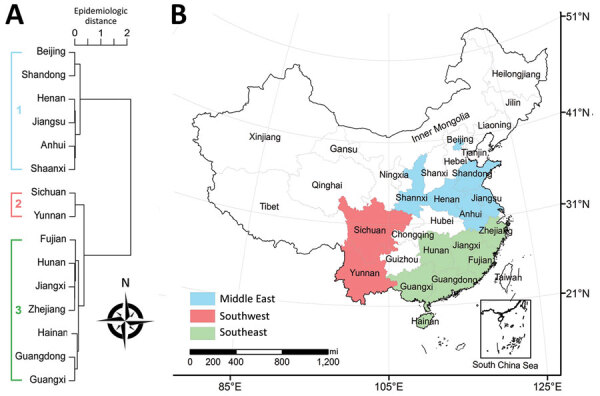
Epidemiologic regions of scrub typhus in China, 2006–2016. A) Epidemiologic regions based on hierarchical clustering, using the Pearson correlation coefficient matrix between average weekly scrub typhus time series of paired provinces that had a cumulative number of cases >100 in 2006–2016 combined. B) Map of identified epidemiologic regions identified by hierarchical clustering), e.g., middle-east (latitude range 31°–41°N and longitude range 105°–125°E), southwest (latitude range 21°–31°N and longitude range 95°–105°E), southeast of China (latitude range 21°–31°N and longitude range 105°–125°E). Other provinces had a combined total of <100 cases in 2006–2016.

During 2006–2016, nearly all cases (93,187/93,481; 99.7%) occurred in the 15 provinces that had a cumulative case count >100. Among these provinces, we identified 3 epidemiologic regions using hierarchical cluster analysis (Figure 4, panels A, B): middle-east (latitude range 31°–41°N, longitude range 105°–125°E); southwest (latitude range 21°–31°N, longitude range 95°–105°E); and southeast China (latitude range 21°–31°N, longitude range 105°–125°E).

### Seasonal Pattern

**Figure 5:**
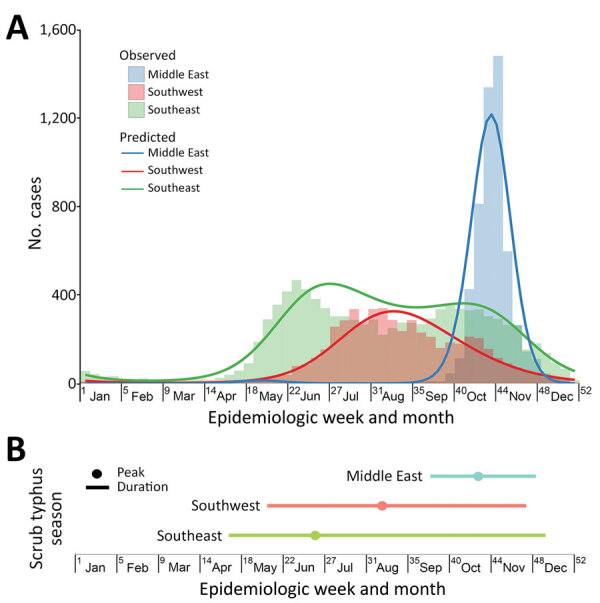
Seasonal characteristics of scrub typhus in China, 2006–2016. A) The weekly number of scrub typhus in year 2016, with a fitted seasonal curve superimposed, by epidemiologic regions identified. B) Duration and peak of scrub typhus seasons in epidemiologic regions. Scrub typhus season was defined as starting with the week in which a fast increase of average weekly case numbers began and ending with the week that the cumulative case numbers captured in the interval between the start and end accounted for >95% of the reported cases in 2006–2016. The colored dot in each region indicates the week with the highest predicted number of scrub typhus cases in the fitted seasonal curve (the peak). The colored bar indicates the number of weeks between season start and end (duration).

Nationally, the seasonal pattern of scrub typhus incidence begins to increase rapidly in May, reaches a peak in October, and falls in November; 94% of cases occurred from May through November in both periods, 1980–1989 and 2006–2016. Heat maps showed consistent seasonal patterns of scrub typhus within each province and between the 2 periods of 1980–1989 and 2006–2016 (Appendix Figure 3), but we observed geographic diversity of seasonal patterns across China. Multiple linear regression models of time series data confirmed and quantified seasonal characteristics of scrub typhus activity in the 3 epidemiologic regions (p<0.001 for all) (Appendix Tables 6, 7). Specifically, the scrub typhus season in the southeast extended from calendar week 17 (early May) to calendar week 50 (mid-December), lasting for 34 weeks and capturing 95% of the total reported cases from this region. In the southwest, the season extended from calendar week 21 (late May) to calendar week 48 (early December), for 28 weeks of duration, capturing 95% of total reported cases in the region. In the middle-east, scrub typhus season lasted 12 weeks, from calendar week 38 (late September) to calendar week 49 (mid-December), capturing 97% of total reported cases in the region (Figure 5, panels A, B).

### Risk for Death

A total of 174 deaths were recorded throughout the country during the 49 years of this study (1952–1989 and 2006–2016). The overall case-fatality ratio was 0.13% (95% CI 0.11%–0.15%); the case fatality ratio from 2006–2016 was significantly lower than that from 1952–1989 (0.06% vs. 0.29%; p<0.001). The following subgroups had a higher risk for death: age >40 years (adjusted odds ratio [aOR] 3.43, 95% CI 1.69–7.72), nonfarmer occupation (aOR 3.56, 95% CI 1.97–6.43), and interval of illness onset to diagnosis >8 days (aOR 2.36, 95% CI 1.07–5.19) (Table 2).

**Table 2:** Risk factors associated with death from scrub typhus, China, 2006–2016

Factor	No. cases	No. deaths	Case-fatality ratio	Crude odds ratio (95% CI)	Adjusted odds ratio (95% CI)*
Sex					
F	49,907	22	0.04	1.00	1.00
M	43,574	35	0.08	**1.82 (1.07–3.11)**	**1.75 (1.02–3.01)**
Age group, y					
<40	25,261	9	0.04	1.00	1.00
40–59	37,660	19	0.05	1.50 (0.68–3.30)	**2.86 (1.22–6.69)**
>60	30,560	29	0.09	**2.70 (1.27–5.72)**	**5.88 (2.55–13.55)**
Residence					
Rural	80,329	37	0.05	1.00	1.00
Urban	11,753	20	0.17	**3.31 (1.92–5.70)**	**1.89 (1.05–3.42)**
Region					
Middle-east	25,186	2	0.01	1.00	1.00
Southeast	45,043	44	0.10	**12.31 (2.98–50.79)**	**10.05 (2.41–41.86)**
Southwest	23,202	11	0.05	**5.97 (1.32–26.95)**	**9.48 (2.05–43.94)**
North and west	50	0	0	NA	NA
Occupation					
Farmer	68,116	24	0.04	1.00	1.00
Nonfarmer	25,365	33	0.13	**3.7 (2.18–6.25)**	**3.56 (1.97–6.43)**
Time from illness onset to diagnosis, d					
<2	20,775	8	0.04	1.00	1.00
2–7	42,875	22	0.05	1.33 (0.59–2.99)	1.67 (0.74–3.77)
>8	29,831	27	0.09	**2.35 (1.07–5.18)**	**2.36 (1·07–5.19)**

*Result from multivariable logistic regression. Boldface type indicates significance (p<0.05). NA, not available.

## Discussion

In this study of a longitudinal surveillance dataset spanning 49 years, we found the occurrence of scrub typhus in China has been maintained at a relatively low level from the 1950s–1980s. However, in the 2000s, incidence rates increased in an unprecedented manner, in both the historically endemic areas and in new areas not previously identified as having cases. In addition, profound epidemiologic changes, including geographic expansion, varying age and sex distribution, and diverse seasonal patterns were further discovered during the period 2006–2016.

In China, some novel natural and socioeconomic factors may have contributed to the increased risk for scrub typhus infection. During the past decade, the reduction of industrial land use, recovering forests and wetlands, and the banning of traditional burning of straw within rural areas have provided a favorable setting for the breeding of rodents and mites, increasing the possibility of human exposure, and the forming of new natural foci for scrub typhus in previously unaffected areas (*29*). In some other scrub typhus–endemic countries, evidence suggests that a combination of climate change and the expansion of humans into previously uninhabited areas may play roles in both the reemergence and the apparent rising incidence of scrub typhus (*30*,*31*).

Globally, the application of improved surveillance mechanisms and enhanced awareness of clinicians have contributed to greater recognition of scrub typhus in some countries, such as India (*1*), South Korea (*1*,*6*), Laos (*6*), and Japan (*3*,*32*). In China, the overall incidence of all notifiable infectious diseases was relatively stable during 2006–2013 (*20*). We believe changes in surveillance may have played a minor role in the increase of scrub typhus in China; although the reincorporating of the disease in the National Notifiable Infectious Disease Reporting Information System and the issuing of 2009 national guidelines may have influenced disease awareness among reporters while the increased use of diagnostic tools such as PCR enabled identification of more cases, the change in reporting alone could not fully explain the drastic changes in the geographic and demographic distribution of scrub typhus. More investigations into the pathogen, the vectors, the environment, and the hosts driving the changing epidemiology of scrub typhus in the country should be conducted.

In this study, we determined that most cases of scrub typhus occur in rural areas and in older persons. During 1990–2020, in rural areas of China, a large number of young and middle-aged male persons have traveled to metropolitan areas seeking better jobs and higher incomes, often leaving their older, younger, or female family members in rural areas. Older family members >60 years of age commonly performed routine farming activities and so are at higher risk for infection. However, the high incidence of scrub typhus cases in patients <5 years of age in 2 specific provinces, Yunnan and Sichuan, should be addressed, and possible risk factors must be further explored.

We discovered that both the number of scrub typhus cases and the number of affected urban areas are rapidly increasing. Because of improved economic and living conditions in China, more persons live in urban areas and travel to rural areas for recreation and entertainment (e.g., fishing, hiking, picnicking, camping, and picking fruit) on the weekends and on holidays. For urban groups susceptible to scrub typhus, frequent outdoor activities in endemic areas increase the risk for infection (*30*,*33*). In 2012, an outbreak of scrub typhus was detected in a city park, implying that the rodent host and mites may inhabit urban settings. In addition, frequent transportation of farmed goods may lead to the migration of rodents carrying infected mites, which may cause the expansion of scrub typhus to more urban and non–typhus-endemic areas (*33*,*34*). 

Our study revealed varying seasonal patterns of the scrub typhus epidemic in China, which have also previously been reported in certain nearby countries. In Japan, 2 types of seasonal patterns peaked in weeks 18–25 (April–May) and weeks 43–52 (November–December) and were located north and south of 37°N latitude (*35*); in South Korea, a single seasonal pattern peaking in October–November was reported (*8*). The appearance of larvae determines the seasonality of scrub typhus. The heterogeneous geographic distribution of *L. deliense* and *L. scutellare* mites may well explain the diverse seasonal patterns of scrub typhus in the southeast, southwest, and middle-east parts of China (*17*). Because of the complex interactions among vectors, hosts, and humans, as well as climatic factors, the seasonal distribution of scrub typhus outbreaks may also vary locally; a spring outbreak of scrub typhus has occurred on an island in Fujian Province (in the southeastern coastal area) during 2000–2005 (*36*). Knowing the exact timing of scrub typhus activity at both national and subnational levels is important to healthcare providers and health officials who use this data to guide diagnostic testing, conduct disease surveillance, plan vector control measures, respond to outbreaks, and provide antimicrobial treatment to febrile patients. Travel precautions for those entering scrub typhus–endemic regions, and strategies for controlling mites, should be given before the beginning of scrub typhus season.

Because scrub typhus is a disease that is treatable with specific antimicrobial medicine, early and accurate diagnosis is essential to reduce the risk for severe complications and death. The case-fatality rate of scrub typhus in China was reduced from 13%–16% to 0.2%–0.4% after the introduction of antirickettsial drugs (*14*). However, the absence of definitive signs and symptoms make difficult the differentiation of scrub typhus from other common febrile diseases, such as murine typhus, typhoid fever, and leptospirosis. Our study reveals that, compared with diagnosis within 2 days after illness onset, diagnosis later than 8 days after the onset of illness poses a 2.36 times higher risk of death for the patient. 

The immunofluorescence assay is the reference diagnostic test for scrub typhus recommended by the World Health Organization (*37*), but the need for standardization has hampered the method’s widespread use. PCR assay targeting on the gene encoding the 56-kDa type-specific antigen is highly sensitive and useful (*38*,*39*) but also has limited uses because of the lack of standardized reagents or commercial diagnostic kits. Currently, the Weil-Felix test, despite its lack of sensitivity and specificity (*40*), is widely accessible throughout China, specifically in conjunction with a previous exposure history or specific eschars of scrub typhus (*41*). The finding that delayed diagnosis is associated with death highlights the need for research and development of inexpensive, accurate point-of-care diagnostic tests for the acute phase of infection in clinical settings.

Our study has 3 limitations. First, we collected all study data used from a passive surveillance system and, therefore, data quality may be influenced by the availability of diagnostic technology, underreporting, and the completeness and accuracy of the data over the years. Diagnosis and notification of cases may vary across areas, affecting the geographic comparisons of incidence. Second, individual case data with demographic information were not reported from 1952–1989; therefore, we could only analyze population characteristics, case distributions, and death risk factors from the period 2006–2016. Furthermore, because scrub typhus was removed from the notifiable disease list in 1990, the lack of surveillance data during 1990–2005 limits the consecutiveness of epidemic trend analysis. Third, data on *O. tsutsugamushi* strains and on the accurate distribution of *Leptotrombidium* mite species were unavailable in this study, making it impossible for us to present the whole picture of scrub typhus in China involving human cases, pathogens, hosts, and vectors. However, data used in this study were the most comprehensive and reliable data on scrub typhus available at national and subnational levels in China; these nationwide report data demonstrate striking changes in epidemiologic features of scrub typhus in China, highlighting the need to conduct further high-quality investigations to better interpret the findings from passive surveillance data.

Among the dozens of countries with endemic scrub typhus, China is one of the few that has established a nationwide surveillance system. By using these long-term incidence data, we thoroughly described the epidemiologic change of scrub typhus over time; our findings are a starting point for further studies to explore additional information related to the global disease burden of scrub typhus. This study may also benefit other scrub typhus–endemic regions outside of China; our findings suggest the possibility of similar epidemiologic changes in areas with similar social and ecologic environments.

As a whole, after ≈40 years of low-level transmission, scrub typhus has become a markedly greater threat in China than previously understood, warranting a higher degree of scrutiny and study to inform health policy. Furthermore, the epidemiologic changes resulting from geographic expansion, demographic transition, and multiple seasonal patterns highlight the need to adjust and enhance current disease prevention and control strategies at national and subnational levels.

Appendix Additional information about scrub typhus in China, 1952–2016.
