# Supplementary material for: Epidemiologic Changes of Scrub Typhus in China, 1952–2016
Source: Emerg Infect Dis. 2020 Jun;26(6):1091–101. doi: 10.3201/eid2606.191168 (PMC7258452; doi:10.3201/eid2606.191168)
Supplement: Appendix — Additional information about scrub typhus in China, 1952–2016. [file 19-1168-Techapp-s1.pdf]

Article DOI: https://doi.org/10.3201/eid2606.191168

Epidemiologic Changes of Scrub Typhus in China, 1952–2016

Appendix

Characteristics of Scrub Typhus Seasonality

To characterize the seasonality of scrub typhus, we used the Poisson regression model to estimate the peak timing and amplitude of the annual and semiannual periodicities of scrub typhus. The generic model formula takes the following form, as described previously (*1–3*).

$$\log(Y_i) = a + b * cos\left(2\pi * \frac{w_i}{52.17}\right) + c * \sin\left(2\pi * \frac{w_i}{52.17}\right) + d * cos\left(4\pi * \frac{w_i}{52.17}\right) + e * \sin\left(4\pi * \frac{w_i}{52.17}\right)$$

where Y_i are the weekly number of scrub typhus cases during 2006–2016 aggregated by epidemiological regions, which were log transformed. w_i is a running index for week, and a, b, c, d, and e are the intercept and seasonal terms to be estimated from the data.

Specifically,

the amplitude of the annual periodicity is estimated as AnnAmpi=sqrt(b^2+c^2);

the shift of the annual periodicity is estimated as AnnPeakTiming=-atan(c/b);

the amplitude of the semi-annual periodicity is estimated as SemiAnnAmpi=sqrt(d^2+e^2); and

the shift of the semi-annual periodicity is estimated as SemiAnnPeakTiming=-atan(d/e).

Appendix Table 1. Summary of laws or regulations related to scrub typhus surveillance and control, mainland China, 1952–2016

ID	Title	Approved by	Issued by	Period enforced	Notes
1	The Administrative Measures of Infectious Disease	The State Council, PRC, on 1955 Jun 1	The Ministry of Health, PRC on 1955 Jul 5	1955 Jul 5–1978 Sep 19	Scrub typhus was one of the 18 notifiable infectious diseases
2	The Administrative Regulation of Acute Infectious Disease	The State Council, PRC	The Ministry of Health, PRC on 1978 Sep 20	1978 Sep 20	Scrub typhus was one of the 25 notifiable infectious diseases
3	The Law on Infectious Disease Control and Prevention	The Standing Committee of the National People's Congress, PRC, on 1989 Feb 21 and revised on 2004 Aug 28		1989 Sep 1–2004 Nov 30; the revised version during 2004 Dec 1–2009 Jan. 3	Scrub typhus was removed from the list of notifiable diseases
4	National Scrub Typhus Control and Prevention Guideline	Issued by Chinese Center for Disease Control and Prevention on 2009 Jan. 4		2009 Jan. 4–current	Scrub typhus was required to be reported in the National Notifiable Infectious Disease Reporting Information System (NNIDRIS), at the Chinese Centers for Disease Control and Prevention (China CDC)

Appendix Table 2. Summary of diagnosis criteria and classification for scrub typhus, mainland China

Variable	1952–2008	2009–present
Criteria or guidelines	Educational Book on Infectious Diseases (First–eighth edition)	National Scrub Typhus Control and Prevention Guideline. 2009
Issued by	Beijing: People's Medical Publishing House	Chinese Center for Disease Control and Prevention
Epidemiology linkage	1.1 An individual who experienced possible outdoor exposure to mite bites three weeks before the onset of illness, i.e., farming, fishing, camping, and straw collection, during the epidemic season of the disease	
Clinical description	2.1 Sudden high fever accompanied by characteristic eschar or ulcer.<br>2.2 Enlarged lymph nodes, skin rash, splenomegaly, or hepatomegaly.	2.1 Fever<br>2.2 Lymphadenopathy<br>2.3 Skin rash<br>2.4 Specific eschars/ulcers
Laboratory tests	3.1 An agglutination titer ≥1:160 in the Weil-Felix test using the OXK strain of *Proteus mirabilis*.<br>3.2 Seroconversion or a four-fold or greater rise in serum IgG antibody titers between acute and convalescent sera detected by using mixed Gilliam, Karp, Kato, and Kawasaki strains of *O. tsutsugamushi* as diagnostic antigen in indirect immunofluorescence antibody assay (IFA).<br>3.3 The detection of O. *tsutsugamushi* 56-kDa gene by polymerase chain reaction in clinical specimens.<br>3.4 The isolation of O. *tsutsugamushi* from clinical specimens.	
Diagnosis and classification	Probable or confirmed cases: a patient with any 3 items among 1.1, 2.1, 2.2, 3.1, 3.2.<br>The case was not further classified as probable or confirmed cases.	Suspected case:<br>(1) a patient with item 1.1, 2.1, plus either 2.2 or 2.3, and was excluded from other diseases. Or<br>(2) a patient with item 2.1, 2.2 and 2.3 during the local epidemic season of scrub typhus (May–November south of Yangtze River and October–November in north of Yangtze River).<br>Probable case:<br>(1) a suspected case with item 2.4. or<br>(2) a patient with item of 1.1, 2.1, and 2.4.<br>Confirmed case:<br>(1) a probable case with any one of the item 3.1–3.4. or<br>(2) a suspected case with any one of the item 3.2–3.4

Appendix Table 3. Variables in the aggregated dataset of scrub typhus cases in China, 1952–1989*

Variables*	Aggregated	Period
No. cases and no. deaths	By month at national level	1952–1989
	By year at provincial level	1952–1979
	By month at provincial level	1980–1989
Annual incidence rate†	At national level	1952–1989
	At provincial level	1952–1989
Annual mortality rate†	At national level	1952–1989
	At provincial level	1952–1989

*The data were aggregated and reported monthly by each province in China, 1952–1989.

†Per 100,000 population

Appendix Table 4. Variables in the individual dataset of scrub typhus cases in China, 2006-2016*

Variables	Definition/Classification	Completeness
Identification	A unique 8-digital number for each case	100% reported
Sex	Male and female	100% reported
Age	Interval from the date of birth to onset date of illness	100% reported
Zone code of residence address	Unique 8-digital number at county and township level	100% reported
Occupation	Occupation/status of case patients	100% reported
Case classification	Suspected case	100% reported
	Probable case	
	Confirmed case	
Date of illness onset	Date of illness onset	100% reported
Date of diagnosis	Date of diagnosis as a probable or confirmed case	100% reported
Date of report	First date of reporting to surveillance system	100% reported
Date of death	Date of case-patient death, if applicable	100% reported

*Data were reported by doctors within 24 hours after diagnosis to the online National Notifiable Infectious Disease Reporting Information System (NNIDRIS) since 2004. NNIDRIS enables all the healthcare institutes across the country to report individual cases of scrub typhus rapidly through the internet to the data center located in the Chinese Center for Disease Control and Prevention.

Appendix Table 5. Geographic information for provinces in China

Province	Inland or coastal	Region	Capital city	Latitude, °	Longitude, °
Heilongjiang	Inland	North and west	Harbin	45.45	126.41
Xinjiang	Inland	North and west	Urumqi	43.79	87.57
Jilin	Inland	North and west	Changchun	43.52	125.19
Liaoning	Coastal	Middle-east	Shenyang	41.50	123.24
Inner Mongolia	Inland	North and west	Hohhot	40.49	111.48
Beijing	Inland	Middle-east	Beijing	39.54	116.28
Tianjin	Coastal	Middle-east	Tianjin	39.09	117.11
Ningxia	Inland	North and west	Yinchuan	38.20	106.16
Hebei	Coastal	Middle-east	Shijiazhuang	38.02	114.28
Shanxi	Inland	Middle-east	Tanyuan	37.52	112.34
Qinghai	Inland	North and west	Xining	36.38	101.45
Shandong	Coastal	Middle-east	Jinan	36.38	117.00
Gansu	Inland	North and west	Lanzhou	36.03	103.49
Henan	Inland	Middle-east	Zhengzhou	34.48	113.42
Shaanxi	Inland	Middle-east	Xi'an	34.16	108.54
Jiangsu	Coastal	Middle-east	Nanjing	32.02	118.50
Anhui	Inland	Middle-east	Hefei	31.51	117.18
Shanghai	Coastal	Southeast	Shanghai	31.14	121.29
Sichuan	Inland	Southwest	Chengdu	30.39	104.05
Hubei	Inland	Southeast	Wuhan	30.37	114.21
Zhejiang	Coastal	Southeast	Hangzhou	30.14	120.09
Tibet	Inland	Southwest	Lhasa	29.40	91.10
Chongqing	Inland	Southeast	Chongqing	29.32	106.32
Jiangxi	Inland	Southeast	Nanchang	28.41	115.52
Hunan	Inland	Southeast	Changsha	28.11	113.00
Guizhou	Inland	Southeast	Guiyang	26.35	106.42
Fujian	Coastal	Southeast	Fuzhou	26.05	119.18
Yunnan	Inland	Southwest	Kunming	25.00	102.41
Guangdong	Coastal	Southeast	Guangzhou	23.08	113.15
Guangxi	Coastal	Southeast	Nanning	22.48	108.20
Hainan	Coastal	Southeast	Haikou	20.02	110.20

Appendix Table 6. Model regression results of scrub typhus seasonality in 3 epidemiological regions of China, 2006–2016*

	Southwest			Southeast			Middle-east		
Item	Model coefficient	SE	p value	Model coefficient	SE	p value	Model coefficient	SE	p value
a	3.684	0.027	<0.001	4.866	0.018	<0.001	1.1539	0.047	<0.001
b	–1.109	0.019	<0.001	–1.068	0.011	<0.001	–0.179	0.068	0.009
c	–1.97	0.026	<0.001	–1.259	0.012	<0.001	–2.852	0.065	<0.001
d	0.243	0.015	<0.001	0.208	0.008	<0.001	–1.286	0.044	<0.001
e	–0.042	0.015	0.005	–0.587	0.009	<0.001	–3.411	0.041	<0.001

*Items a, b, c, d, and e are the intercept and seasonal terms to be estimated from the data. SE, standard error.

Appendix Table 7. Seasonal characteristics of scrub typhus in the 3 epidemiological regions of China, 2006–2016*

Epidemiologic region	Intercept	AnnAmpi	AnnPeakTiming	SemiAnnAmpi	SemiAnnPeakTiming
Southwest China	3.684	2.26	0.332	0.247	0.027
Southeast China	4.866	1.651	0.362	0.623	0.196
Middle-east China	1.1539	2.858	0.26	3.646	0.307

*Values provided are in logarithmic scale. AnnAmpi, amplitude of annual periodicity; AnnPeakTiming, shift of the annual periodicity; SemiAnnAmpi, amplitude of the semi-annual periodicity; SemiAnnPeakTiming, shift of the semiannual periodicity.

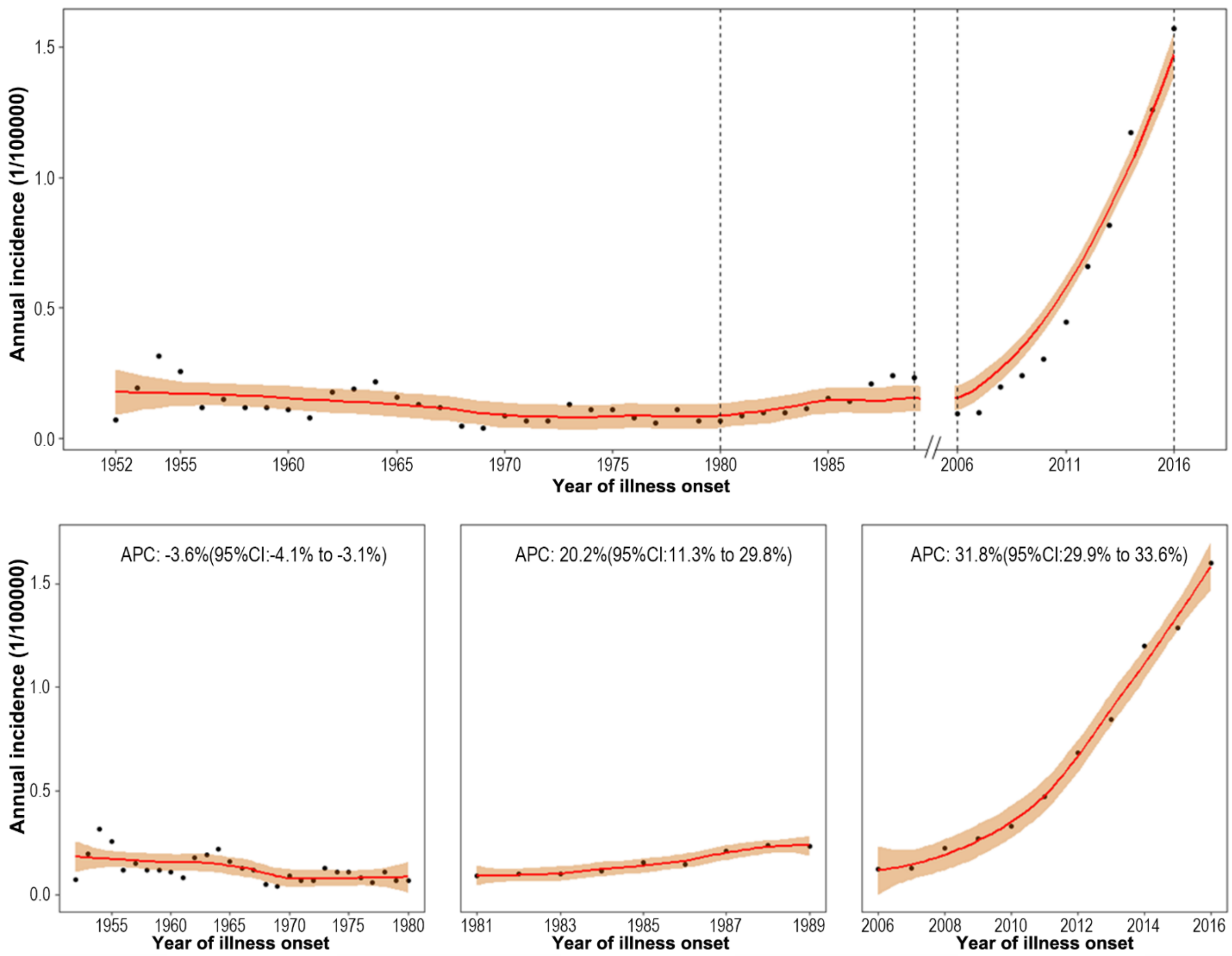

Appendix Figure 1. Temporal trends of incidence rate of scrub typhus in China, 1952–2016. A) Overall trend of overall incidence from 1952–2016. B) Annual percent change (APC) of incidence rate during 1952–1980. C) APC of incidence rate during 1981–1989. (D) APC of incidence rate during 2006–2016. Black dots indicate observed incidence rates; red lines indicates incidence trends; orange shading indicates 95% CI.

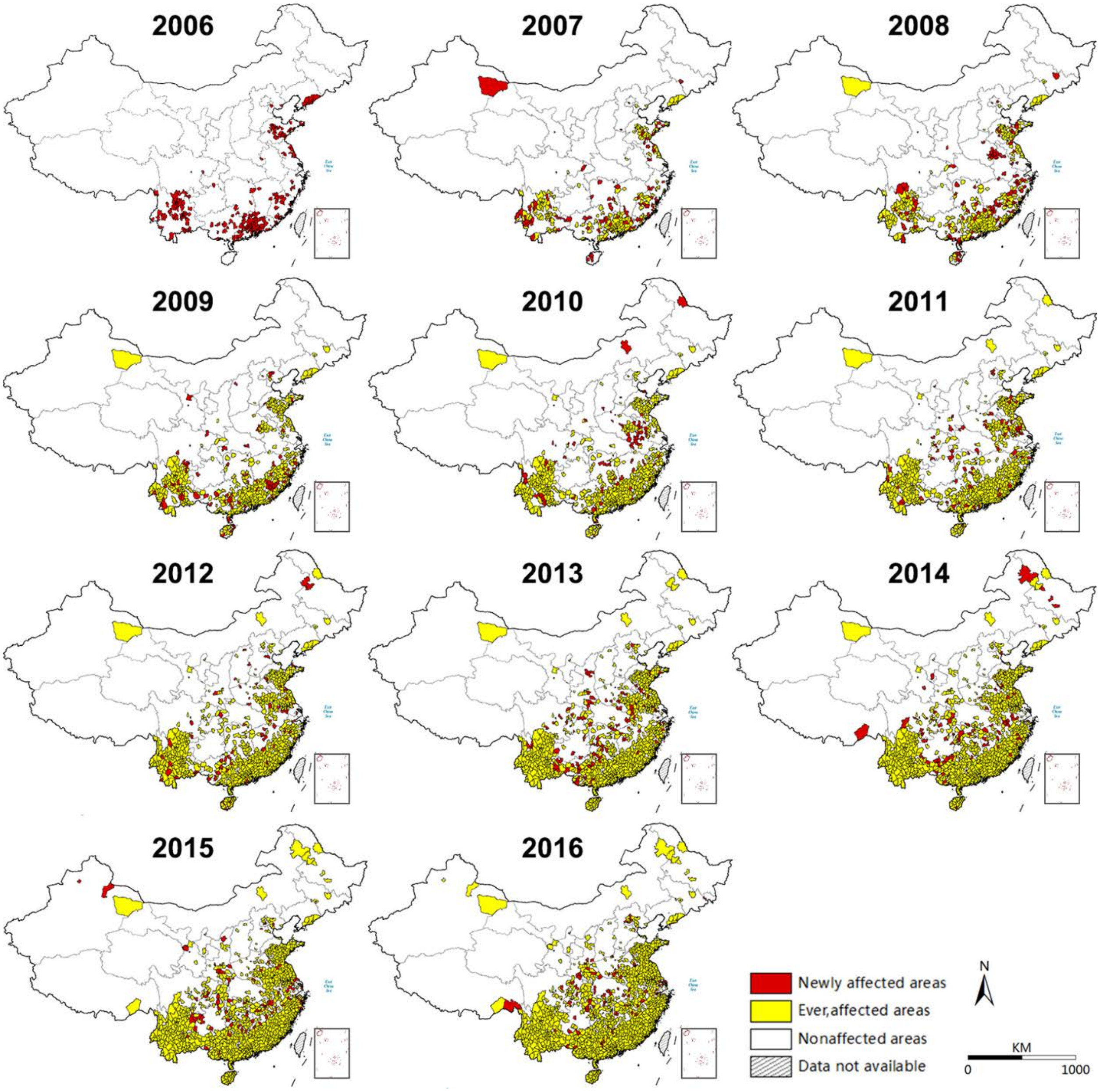

Appendix Figure 2. Geographic expansion of scrub typhus in China, 2006–2016.

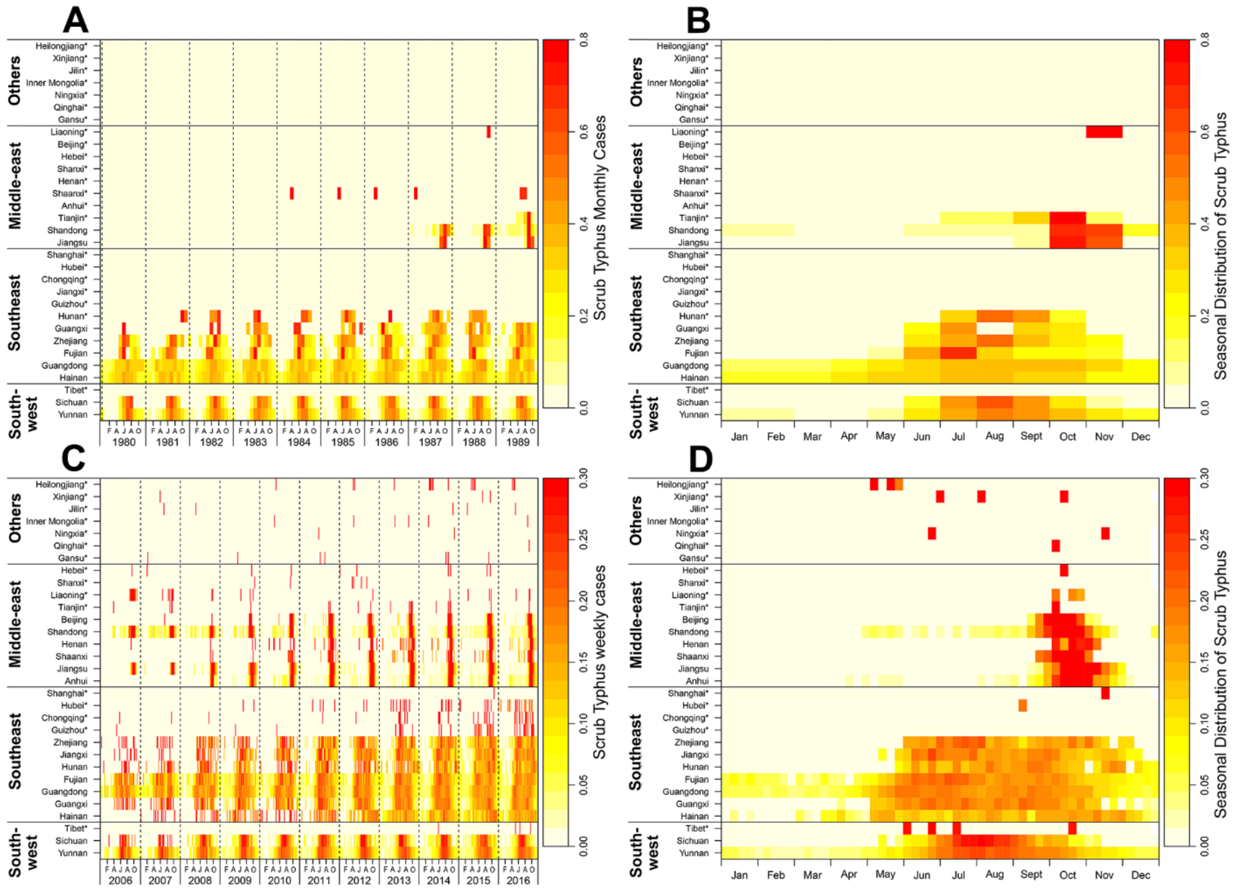

Appendix Figure 3. Heat map of provinces with scrub typhus, by epidemiological region and the latitude of the capital city of each province. A) Time series of monthly cases during 1980–1989, standardized by the annual number of cases reported by each province and standardized to a range of 0–1. B) Seasonal distribution of cases of scrub typhus by province, plotted as the mean value of the proportion of cases in each month of the year from 1980–1989. (C) Time series of weekly cases during 2006–2016, standardized by the annual number of cases reported by each province and standardized to a range of 0–1. (D) Seasonal distribution of cases of scrub typhus by province, plotted as the mean value of the proportion of cases in each week of the year from 2006–2016. Asterisk (*) indicates provinces in which the total number of cases in 2006–2016 combined was <100.

References

<jrn>1. Yu H, Alonso WJ, Feng L, Tan Y, Shu Y, Yang W, et al. Characterization of regional influenza seasonality patterns in China and implications for vaccination strategies: spatio-temporal modeling of surveillance data. PLoS Med. 2013;10:e1001552. PubMed https://doi.org/10.1371/journal.pmed.1001552</jrn>

<jrn>26. Naumova EN, Jagai JS, Matyas B, DeMaria A Jr, MacNeill IB, Griffiths JK. Seasonality in six enterically transmitted diseases and ambient temperature. Epidemiol Infect. 2007;135:281–92. PubMed https://doi.org/10.1017/S0950268806006698</jrn>

<jrn>27. Xing W, Liao Q, Viboud C, Zhang J, Sun J, Wu JT, et al. Hand, foot, and mouth disease in China, 2008–12: an epidemiological study. Lancet Infect Dis. 2014;14:308–18. PubMed https://doi.org/10.1016/S1473-3099(13)70342-6</jrn>
